# Socio-temporal contextual and community factors associated with daily exclusive ENDS use and dual use with tobacco cigarettes among adolescent vapers: an ecological momentary assessment study

**DOI:** 10.1186/s12889-022-14787-1

**Published:** 2022-12-07

**Authors:** Melissa H. Abadi, Stephen R. Shamblen, Kirsten Thompson, Sharon Lipperman-Kreda, Joel Grube, Bonnie O. Richard, Camila Aramburu

**Affiliations:** 1 Pacific Institute for Research and Evaluation, 401 West Main Street, Suite 2100, Louisville, KY 40202 USA; 2 Prevention Research Center, Pacific Institute for Research and Evaluation, 2030 Addison Street, Suite 410, Berkeley, CA 94704-2642 USA; 3 School of Public Health, University of California, Berkeley, USA

**Keywords:** Dual use, ENDS, Tobacco cigarettes, Adolescents, Ecological momentary assessment, Contextual factors, Community factors

## Abstract

**Background:**

Adolescents who dual use ENDS with tobacco cigarettes are more likely to have an increased risk of developing dependence. Yet, little is understood about the factors driving dual use among adolescents. The current study sought to reveal the day-to-day socio-temporal contextual and community factors associated with adolescents’ use of electronic nicotine delivery systems (ENDS), and how these factors predict dual use with tobacco cigarettes.

**Methods:**

We collected ecological momentary assessments (EMA) from a sample of 50 adolescent past two-week vapers (ages 14–17 years old) over 14 days. Daily EMA data were collected on ENDS and tobacco cigarette use, as well as a range of contextual (i.e., motivations to vape, location of vaping, who with when vaping) and community factors (i.e., exposure to peers vaping, to adults vaping, to ENDS advertising, to ENDS warning messages). Our primary analyses were multilevel regressions, accounting for daily observations nested within individuals (*N* = 700 observations).

**Results:**

Participants used ENDS exclusively on 44% of days and dual used ENDS and tobacco cigarettes on 8% of the days. Dual use days (versus exclusive ENDS use days) were associated with “vaping because tobacco use was prohibited” (OR = 34.65, *p* < .05). Also, dual use days (versus no use days) were associated with greater exposure to adults vaping (OR = 5.59, *p* < .05), peers vaping (OR = 7.48, *p* < .05), and (c) ENDS advertisements or promotions (OR = 2.12, *p* < .01), whereas exclusive use days (versus no use days) were only associated with greater exposure to peers vaping (OR = 2.58, *p* < .01).

**Conclusions:**

Results showed that exposure to peers and adults vaping and exposure to ENDS marketing were associated with same day dual use behaviors. And, that adolescents who dual used were motivated to use ENDS because they were easy to conceal. Findings support stricter regulation of ENDS marketing and for smoke-free air laws that include ENDS. In addition, these findings support prioritizing family- and school-based prevention programming that effectively communicates risk associated with ENDS use, including heightened risk of dual use and dependence. Such efforts can reduce the number of adolescents who use ENDS as well as the number who transition to tobacco cigarette use.

## Background

The vast number of available youth-appealing electronic nicotine delivery systems (ENDS) has drastically changed the tobacco landscape and provided many options for adolescents to initiate and continue nicotine use [1–4]. ENDS use among U.S. adolescents hit its peak in 2018 with approximately 4.8 million middle- and high-school students reporting current ENDS use [3]. Prevalence remains high, as 2.06 million adolescents reported current use of ENDS in 2021 [5]. Of concern, adolescent ENDS users are more likely than non-users to initiate tobacco cigarette use [6–8] and become regular smokers [7, 9, 10]. Further, adolescents who dual use ENDS and tobacco cigarettes are less likely than exclusive ENDS users to stop using tobacco products 2–3 years later [11], and more likely to have an increased risk of developing dependence [1, 12, 13]. The overall goal of this study was to examine the differential influences of exclusive ENDS use and dual use with tobacco cigarettes among adolescents.

Dual use of ENDS and tobacco cigarettes among adolescents is an understudied area of research [14, 15]. While older populations may be dual using ENDS and tobacco cigarettes as an effort to reduce or quit tobacco cigarettes, this motivation is less likely among adolescents [14]. In fact, studies have shown that ENDS use among younger populations is associated with more frequent and intensive tobacco cigarette use [16]. It is, therefore, important to understand the factors that may be driving dual use among adolescent ENDS users to inform policies and prevention efforts.

Research indicates that environments favorable of ENDS use influence susceptibility to and use of tobacco cigarettes [8, 10, 17–20]. Studies further show that certain socio-ecological factors may be driving dual use behaviors among adolescents. For example, compared to exclusive ENDS use, dual use among adolescents is more likely to be associated with greater positive smoking expectancies [21], lower parental monitoring [21], lower perceived risk of tobacco use [22, 23], higher exposure to and receptivity to tobacco promotions and marketing [22], and lower receptivity to antismoking ads [23].

This existing research enhances our understanding of how risk factors are differentially associated with exclusive ENDS use and dual use of ENDS and tobacco cigarettes. However, much of the research in this area has not examined the full socio-ecological environment of adolescent ENDS and dual use by considering a wide range of socio-temporal contextual and community factors in one study. Since tobacco use behaviors are driven by the interaction among individual and environmental risk factors within one’s ecological context [24, 25], it is important to consider contextual and community factors together in order to identify factors that can inform prevention and policy efforts [26]. Further, cross-sectional and longitudinal surveys do not capture the daily and momentary factors that influence adolescents’ real-world exclusive ENDS use versus dual use and are subject to recall bias [27].

Ecological momentary assessment (EMA) provides an ideal methodology to reveal a wide range of contextual and community factors associated with adolescent tobacco use behaviors because it involves collecting data close to when behaviors and experiences occur in real-world contexts, reducing recall bias and maximizing ecological validity [28]. EMA studies have demonstrated that certain high-risk ecological contexts are associated with tobacco use behaviors [28]. Various EMA studies have examined use behaviors and associations among young adults, assessing exclusive ENDS use, exclusive tobacco cigarette use [29, 30], or use of any tobacco products [30, 31]. However, EMA studies have not examined exclusive ENDS use and dual use with tobacco cigarettes among adolescents, leaving a gap in knowledge of the day-to-day contextual and community factors that influence adolescents’ dual use of ENDS and tobacco cigarettes. This is a critical area of study, given the established association of ENDS use and subsequent use of tobacco cigarettes among adolescents [6, 8] and the heightened risk of dependence [32].

The purpose of the current study was to reveal the day-to-day contextual and community factors of adolescent ENDS use and how they predict dual use with tobacco cigarettes among adolescents. Based on the social ecological framework [24], and on research that suggests favorable ENDS environments can influence tobacco cigarette use among adolescents [17], the overarching hypothesis of our study was that day-to-day socio-temporal contextual factors (i.e., motivations to vape, location of vaping, who with when vaping) and community factors (i.e., exposure to peers vaping, to adults vaping, to ENDS advertising, to ENDS warning messages) would be associated with same-day exclusive ENDS use and dual use of ENDS and tobacco cigarettes. Based on the exploratory nature of research questions related to which socio-temporal contextual and community factors would be differentially associated with exclusive vaping versus vaping with tobacco cigarettes, we did not make specific hypotheses about these relationships.

## Methods

The study design is described in greater detail elsewhere [33, 34], including more information on sample recruitment and selection as well as study procedures. All study protocols, including obtaining informed consent to participate, were carried out in accordance with relevant guidelines and regulations, and approved by PIRE’s Institutional Review Board (federal-wide assurance: FWA00003078). As participants were all under 18, we obtained parental informed consent and participant assent for all 50 participants.

### Recruitment

Adolescents were recruited to participate in surveys that asked about opinions, perceptions, marketing exposures, and use of e-cigarettes and tobacco through a study website and recruitment flyers. Inclusion criteria were: (a) being between ages 14 and 17 years old, (b) living within a 100-mile radius of Louisville, Kentucky, and (c) self-report past two-week ENDS use (i.e., if they responded “yes” to vaping ENDS in the past two weeks). To maintain confidentiality and avoid potential response bias, neither parents nor the adolescents were informed that past two-week ENDS use was an inclusion criterion. Parental consent and youth assent were obtained electronically.

### Participants

The first 50 eligible adolescents to complete the screening were enrolled. Of those, three did not complete the initial online survey and did not progress to the EMA surveys, and one dropped out of the study on the second day of EMA. Those four adolescents were replaced by the next four eligible adolescents on our recruiting list to obtain a sample of 50 adolescents (to ensure adequate statistical power). Participants were asked to complete EMA surveys over two weeks (14 days). On average, each participant provided 13.4 days of EMA data, resulting in 670 of 700 possible observations for a 96% completion rate.

### Study design

Data were collected January through October 2018. Participants were sent a link via text or e-mail to a 30-min initial online survey that included demographics, individual characteristics, and ENDS and tobacco use behaviors. Next, an online text messaging platform was used to send survey links and reminders to a cell phone provided to participants by the study team. On each of 14 consecutive days, participants were asked to respond to the EMA surveys to report vaping and tobacco use behaviors, reasons for vaping, and contexts of vaping (where, with whom, vaping exposures). Incentives for study participation included: $15 for completing the initial survey, $5 for each completed EMA survey, a $20 bonus if they completed all EMA surveys, and $15 for returning the study phone.

On Mondays through Thursdays, all participants were prompted to complete the survey once daily at 4 pm, asking them about the past 24 h (i.e., from 4 pm the prior day to 4 pm that day). On Fridays, all participants were prompted to complete the survey at 4 pm and 8 pm, reporting on time periods from 4 pm the day prior to 4 pm that day, and from 4 to 8 pm that day. On Saturdays and Sundays, all participants were prompted at 11am, 4 pm, and 8 pm, reporting on time periods from 8 pm the prior day to 11am that day, from 11am to 4 pm that day, and from 4 to 8 pm that day. Data collection was set up this way to capture more data when the adolescents were not in school when we suspected there would be more use (i.e., the weekend). For analysis, we aggregated the weekend datapoints into measures representing 24-h periods to create daily observations.

### Initial survey measures

#### Individual characteristics

Participants were asked to respond to questions on individual and family characteristics including age, sex, and race/ethnicity. Socioeconomic status was assessed through one item asking how much money they had in a typical week to spend on whatever they want, not including basic necessities. The nine response categories were coded and rounded to the next integer to approximate an interval measure by taking the midpoints (< $5 = $4; $5-$10 = $8; $11 to $25 = $19; $26 to $50 = $39; $51 to $75 = $64; $76 to $100 = $89; $101 to $125 = $114; $126 to $150 = $139; and > $150 = $151).

### Daily measures

#### ENDS use and dual use with tobacco cigarettes

Each survey asked adolescents to report if they vaped nicotine during the survey window. If they said yes, they were asked how many times they vaped nicotine (i.e., number of occasions) during the survey window and how many total puffs they typically vaped on each occasion. To create daily vaping measures on weekend days, we summed use occasions across all survey time windows to equal a 24-h period. The total number of vaping puffs per day was estimated as the product of the sum of use occasions and the mean of typical puffs per day. At the 4 pm survey each day, participants were also asked if they smoked tobacco cigarettes during the survey window, and a dual use outcome was created representing use of tobacco cigarettes and ENDS on the same day. We coded outcome measures to represent (a) dual use, (b) ENDS use only, (c) cigarette use only, or (d) neither. We coded cigarette use only occasions as missing data in our substantive analysis, due to the small number of observations (*n* = 56).

#### Contextual factors of ENDS use

If adolescents said yes to vaping nicotine during a survey window, they were asked a series of questions about the last time they vaped nicotine, including: *who they vaped with* (recoded to by myself versus with others), *where they vaped* (recoded to someone’s home, my home, outdoors, or school), and *why they vaped* on the last occasion (recoded to it’s easy to get, I like the flavors, it feels good, trying to quit tobacco cigarettes, no odor, or because tobacco is prohibited). Since weekend data were aggregated across multiple observations to the daily level, we selected context responses from the period between the last vaping occasion on the prior day at 4 pm to the current day 4 pm to make these observations consistent with weekday observations.

#### Community factors of ENDS use

At the 4 pm survey each day, participants were asked if, in the last 24 h (i.e., 4 pm to 4 pm), they were exposed to others’ use, warning messages about vaping, and advertisements about vaping. For *exposure to others’ use*, participants were asked if they saw any adults (yes, no) or people their age (yes, no) using e-cigarettes or vape devices. For *exposure to warning messages*, they were asked whether they saw or heard any health warning messages about vaping (yes, no). For *exposure to ads*, they were asked whether they saw e-cigarettes or vape devices in various types of media (i.e., inside or outside of a store or on a billboard in or near your neighborhood, inside or outside of a store or on a billboard in or near your school, online or social media, and in a magazine/TV/movie). Responses for ad exposure were re-coded into an overall daily exposure to ENDS advertising variable (0–4 exposures).

### Analysis

Given the focus of our study on socio-temporal contextual and community factors related to ENDS use and dual use of ENDS with tobacco cigarettes, our analyses excluded days on which only tobacco cigarette use occurred(*n* = 56). Our primary analyses were random intercept, multilevel, binomial and multinomial logit regressions, accounting for daily observations nested within individuals. Binomial models were used for contextual factors since these measures were only asked when adolescents reported ENDS use (either exclusive or dual use), and multinomial models were used for community factor predictors. Specifically, binomial models examined exclusive ENDS use (referent category) vs. dual use, and our multinomial models examined exclusive ENDS use, dual use of ENDS and tobacco cigarettes, and no ENDS or tobacco cigarette use (referent category). There was a great deal of variability among participants (i.e., daily changes in use patterns occurred for participants), as evidenced by large intraclass correlation coefficients (ICC) (see Table 1). Substantive models regressed outcome on predictors, where predictors were grouped into topical areas (i.e., where vaped, why vaped, who with when vaped, exposure to adults vaping, exposure to peers vaping, warnings, and advertising) and we ran separate models for each topical area. Odds ratios and their accompanying 95% confidence intervals were calculated for models. Binomial models were fit using the lmer library [35] in the R environment for statistical computing [36] and multinomial models were fit using Stata 15 [37].

**Table 1:** Percentages, means (and standard deviations) of sample characteristics

Sample Sizes	
Individuals	50
Observations	670
Demographics	
Age	16.22 (.86)
Male	42%
White race	90%
Spending money / week	$52.08 ($49.47)
Type of Use (EMA)	
Exclusive ENDS use days (ICC^*^ = .51)	44%
Dual use days (ICC^*^ = .52)	8%

Note: All means and percentages based on 50 participants, except type of use, which is based on 670 observations

^*^ICC indicates the intraclass correlation coefficient

## Results

### Participant characteristics

Table 1 shows participant characteristics. They had a mean age of 16.2 years (SD = 0.86). Close to half of the sample was male (42%). The majority of the sample reported being non-Hispanic White (90%). Participants reported having between $4 and $151 (quartiles: $19, $29, and $89) per week as spending money.

### Patterns of daily nicotine use

Participants used ENDS exclusively on 44% of days and dual used ENDS and tobacco cigarettes on 8% of days. Considering all possible daily outcomes, participants neither used ENDS nor tobacco cigarettes on 39% of days. Considering the global vaping patterns of individuals over the entire 14 days, 66% (n = 33) of participants had exclusive ENDS use on all use days, 14% (*n* = 7) had dual use on all use days, and 16% (n = 8) had a mix of exclusive ENDS and dual use days.

### Day-to-day contextual factors associated with dual use of ENDS and tobacco cigarettes versus exclusive ENDS

Table 2 shows results of the binomial logistic regressions assessing associations of contextual factors with daily dual use of ENDS and tobacco cigarettes, relative to exclusive ENDS use. Controlling for covariates, dual use (versus exclusive ENDS use) was positively associated with “vaping because tobacco use was prohibited” (OR = 34.65, *p* < 0.05). Also, dual use was marginally associated with ENDS not having an odor (OR = 16.33, *p* = 0.07) and not being at one’s own home (OR = 0.10, *p* = 0.09).

**Table 2:** Time variant predictors of exclusive ENDS vs dual use days among adolescents for contextual factors (*n*_*days*_ = *337)*

	**OR**	**95% CI**	
*Where Vaped*			
someone's home	.52	.05, 5.78	
my home	.10	.01, 1.46	^+^
Outdoors	1.46	.08, 25.33	
School	1.50	.08, 29.41	
*Why Vaped*			
easy to get	.33	.04, 2.83	
like the flavors	4.81	.40, 57.57	
feels good	.19	.02, 1.84	
want to quit cigs	.60	.04, 8.96	
no odor	16.33	.78, 341.11	^+^
tobacco is prohibited	34.65	2.29, 523.26	^*^
*Who with when Vaped (Others vs. Alone)*			
vaped with others	1.02	.21, 4.88	

^***^p < .001

^**^p < .01

^*^ *p* < .05

^+^*p* < .10; tests included sex, age, and income as covariates

### Day-to-day community factors associated with exclusive ENDS use and dual use with tobacco cigarettes versus no use

Table 3 shows results of the multinomial logistic regressions assessing associations of contextual factors with daily exclusive ENDS use or dual use of ENDS and tobacco cigarettes, relative to no use. Controlling for covariates, exclusive ENDS use (versus no use) was associated with greater exposure to peers vaping nicotine (OR = 2.58, *p* < 0.01) on each day. Focusing on dual use of ENDS and tobacco cigarettes, controlling for covariates, days when adolescents reported dual use of ENDS and tobacco cigarettes (versus no use), were predicted by (a) greater exposure to adults vaping (OR = 5.59, *p* < 0.05), (b) greater exposure to peers vaping (OR = 7.48, *p* < 0.05), and (c) greater exposure to ENDS advertisements or promotions (OR = 2.12, *p* < 0.01).

**Table 3:** Time variant predictors of exclusive ENDS days and dual use days (vs. no use) among adolescents for community factors (*n*_*days*_ = *587)*

	**Dual Use Days vs. No Use Days**			**Exclusive Use Days vs. No Use Days**		
	**OR**	**95% CI**		**OR**	**95% CI**	
*Exposure to Adults Vaping*	5.59	1.40, 22.29	^*^	.78	.40, 1.52	
*Exposure to Peers Vaping*	7.48	1.31, 42.76	^*^	2.58	1.44, 4.64	^**^
*Exposure to Warnings*	1.49	.33, 6.63		1.72	.76, 3.86	
*Exposure to Advertising*	2.12	1.22, 3.71	^**^	.96	.73, 1.26	

^***^ *p* < .001

^**^ *p* < .01

^*^ *p* < .05

^+^ *p* < .10; tests included sex, age, and income as covariates. Each row represents a separate model run as a multinomial logit model

## Discussion

Our study examined exclusive ENDS use and dual use with tobacco cigarettes among adolescents through EMA methods to identify day-to-day socio-temporal contextual and community factors that may uniquely influence these behaviors. Given the persistent prevalence of ENDS use among adolescents [5] and studies which show that ENDS users are more likely to subsequently initiate combustible tobacco [6], it is critically important to understand the factors that may be driving dual use among adolescent vapers. The adolescents in our sample used nicotine consistently, reporting nicotine use on more than half of the days within the two-week EMA study period. They were using ENDS exclusively on 44% of those days and dual using ENDS and tobacco cigarettes on 8%. The frequency of nicotine use within a two-week period is concerning, especially given research that shows experimenting with ENDS and multiple other tobacco products before 18 years old is strongly associated with subsequent daily cigarette smoking [38]. Given these concerns, it is imperative to identify risk factors of dual use of ENDS and tobacco cigarettes to inform comprehensive prevention and policy efforts that address multiple tobacco products.

The results of our study suggest that there are certain socio-temporal contextual and community factors that predict dual use days among adolescent vapers. Indeed, days where adolescents dual used ENDS and tobacco cigarettes were predicted by vaping motivations related to being able to conceal use. Specifically, dual use days were predicted by the motivation of vaping because tobacco use is prohibited. This finding supports other research that suggests that day-to-day contexts and situations drive nicotine product choice [29, 30]. Our study extends these findings by demonstrating that even among adolescents who dual use, product choice might be driven by motivations related to their need to continue using nicotine, even in situations where tobacco cigarettes are unacceptable [29, 39, 40]. Importantly, our study did not find any significant associations between ENDS use and motivations to quit. Together, these findings might suggest that adolescents who regularly use ENDS are dual using with ENDS to use nicotine more often, not less [32].

Results showed that dual use days and exclusive ENDS use days were both predicted by greater exposure to peers vaping on that day. Adolescents may be influenced by peer ENDS users to engage in both vaping and smoking behaviors due to social pressure or social access (i.e., the social nature of vaping and smoking, sharing of devices and cigarettes, and increased access when with peers) [41, 42]. In addition, research among young adults shows that exposure to ENDS use increases the desire for both ENDS and combustible tobacco cigarettes [43], so that is also a plausible explanation, although we did not assess craving or dependence in this study. Future research should examine how exclusive and dual use contexts among adolescents vary by individual craving and dependence symptoms.

Same day exposure to adults vaping predicted dual use but not exclusive ENDS use. These results may reflect adolescents’ social access to tobacco cigarettes from adults in their environment who use ENDS, as well as the strong influence of favorable tobacco norms among older persons in adolescents’ environments [18, 44]. Similar to our findings on peer vaping, this could also reflect the influence of exposure to nicotine use on the desire for both ENDS and combustible tobacco cigarettes [43].

Dual use days were also predicted by same-day exposure to ENDS promotions and advertising. These results are supported by another EMA study that found that adolescent participants were more likely to report recent tobacco use if they also reported recent tobacco marketing exposure [31]. Our study extends this research by showing that ENDS-specific marketing exposure may impact same-day dual use of ENDS and tobacco cigarettes. This finding has important implications since ENDS marketing is pervasive and includes youth-appealing marketing channels and content [45–47]. In addition, ENDS marketing does not have the comprehensive restrictions that have been imposed on the marketing of traditional tobacco products [48, 49].

Interestingly, exposure to warning messages about ENDS was not associated with exclusive or dual use behaviors. This could be explained by research that found that warning labels on ENDS vials did not grab the attention of the young adults nor influence the perceived addictiveness of ENDS [50]. The researchers suggest that the warning labels on vials have limited potential impact in competition with the other attractive packaging. In addition, it is possible that our sample did not even see the warning labels or messaging on the ENDS packaging or on the e-liquid vials due to the common practice of sharing vaping devices among adolescents. Additional research is needed to determine what types of warning messages and labels can counteract youth-appealing product packaging as well as the pervasive marketing tactics that influence tobacco use.

### Strengths and limitations

One of the strengths of this study is that it allowed us to measure exclusive ENDS use and dual use with tobacco cigarettes with a method that reduces recall bias compared to cross-sectional or more typical longitudinal survey methods that rely on past 30-day and past-year recall measures. While EMA methods provide more precise measurement and allow for an examination of secular trends in use, costs usually dictate that fewer participants are followed using these methods. Our study had 50 participants, which was sufficient to examine within-participant variability, but proved insufficient for examining between-participant variability. More specifically, 50 participants did not yield sufficient variability between participants and led to multicollinearity for within and between predictors (i.e., rs ranged from 0.54 to 0.90, median = 0.71), which often led to poor fitting models that did not converge. Nevertheless, as the primary focus of this paper is on the patterns of variability on repeated observations, the sample size is 700 (50 adolescents × 14 days), which is sufficient. And, while statistical power does become a concern with reduced sample size, we suspect this is not the case, given we found a consistent pattern of results across dependent measures. Finally, since we drew our sample from a small catchment area in Kentucky, we are unable to make inferences about the general population of adolescent ENDS users. Future studies with larger, more representative samples need to be conducted to see if these findings can be replicated and extended.

## Conclusions

Our study advances the extant research on adolescent ENDS use by examining a unique set of socio-temporal contextual and community factors that can be prime targets for policy and prevention efforts aimed at reducing the number of adolescents who transition to tobacco cigarette use. Overall, findings support research that shows that favorable ENDS environments predict both vaping and smoking behaviors among adolescents. However, our study also demonstrates the strong influence of exposure to peers and adults vaping and to ENDS marketing on adolescent dual use behaviors that same day. In addition, our findings show how characteristics of ENDS (e.g., easy to conceal) make it possible for adolescents to use nicotine on a more regular basis and in various settings that would not be possible with tobacco cigarettes. Together, these findings support stricter regulation of ENDS marketing and enforcement of smoke-free air laws that include ENDS. In addition, family- and school-based prevention programming should focus on communicating the risks of ENDS use, including heightened risk of subsequent tobacco cigarette use and dependence. Finally, given that the results of our study suggest that this young group of vapers is seeking opportunities for nicotine and using multiple products to get it, future EMA research should assess the associations of exclusive and multiple product use on dependency and withdrawal symptoms among this young population. To better understand these important issues among youth, future research should involve larger samples of adolescent ENDS users and additional assessments of dual and poly use of tobacco products, including novel tobacco products (e.g., heat-not-burn products, nicotine pouches, etc.).

## Data Availability

The data supporting this article are available from the corresponding author, MA, upon reasonable request.

## Abbreviations

ENDS: Electronic nicotine delivery systems
EMA: Ecological momentary assessment
ICC: Interclass correlation
OR: Odds ratio
